# Status determinants, social incongruity and economic transition: Gender, relative material wealth and heterogeneity in the cultural lifestyle of forager-horticulturalists

**DOI:** 10.1371/journal.pone.0220432

**Published:** 2019-09-03

**Authors:** Alan Frank Schultz

**Affiliations:** Department of Anthropology, Baylor University, Waco, Texas, United States of America; University of Richmond, UNITED STATES

## Abstract

For small-scale societies, transitions from self-sufficiency to cash-based labor in market economies have been associated with the exacerbation of existing, and the emergence of new, social incongruities. Social incongruity occurs when two or more of a person’s status determinants (e.g. age, gender, wealth) conflict, resulting in reduced social status. A central focus of theory and research on social incongruity is the relationship between the cultural prototype of what is needed to live a good life–or lifestyle–and status determinants. Assessment of status determinants is challenging because of their relative nature at multiple levels of analysis. This study uses theory and methods from cognitive anthropology to investigate whether and how individual knowledge of a cultural lifestyle prototype conflicts with status determinants at two levels of economic transition among 101 adults from a small-scale society of forager-horticulturalists in Bolivian Amazonia, the Tsimane’. Results support cultural consensus in a 38-item model labeled *market* lifestyle (explaining 72.7% of sample variance). While the model includes both overlapping traditional (e.g. weaving) and market-related (e.g. education) items and behaviors, most market alternatives were rated higher. When market lifestyle was tested for social incongruity against other status determinants, only gender predicted variation. Thematically, when lifestyle was stratified by gender, men rated several items of relational wealth higher than women did. Analysis of model residual agreement revealed heterogeneity in the form of a *syncretic* lifestyle model (explaining 18.2% of additional variance). Participants whose knowledge better matched syncretic lifestyle rated traditional items and market alternatives closer to parity. Agreement with the syncretic model correlated with lower material wealth and less market integration. In sum, the findings document a modern, market-oriented form of Tsimane’ lifestyle that varies ontologically from past modelling and ethnographic accounts in preferred forms of livelihood and wealth.

## Introduction

**Fig 1:**
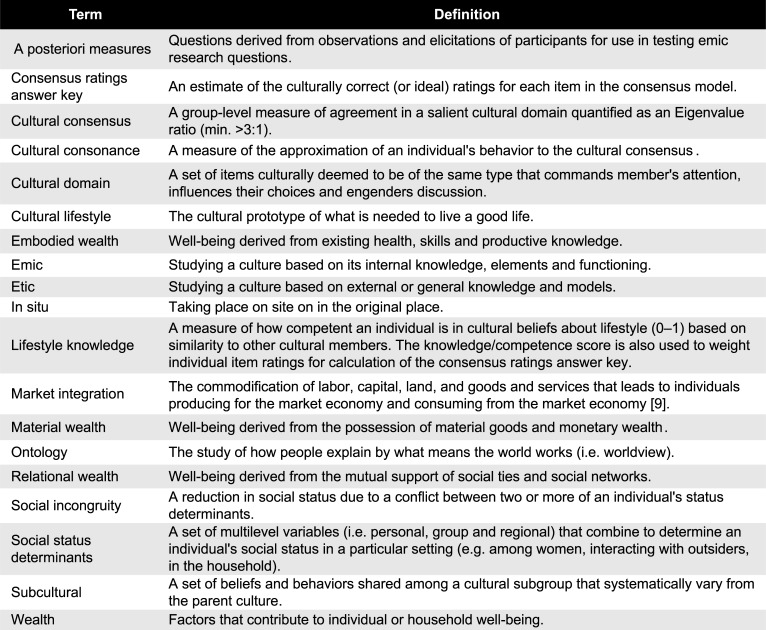
Glossary of key terms.

Research on economic transitions has long recognized that the process influences, and is influenced by, several cultural domains including family life, social support, material consumption and lifestyle [1–6]. Cultural changes due to economic transition can be especially profound during early-stage market integration, wherein a self-sufficient, small-scale society merges with a cash-based market system [7,8]. These wide-ranging effects include alteration to shared concepts of wealth and value [9] through the “commodification of labor, capital, land, and goods and services [that leads to] individuals producing for the market and consuming from the market” (p.594). As change takes hold, the existing social order is disrupted as the hierarchy of determinants that contribute to social status is rearranged (e.g. formal education begins to be valued in addition to traditional skills, or monetary wealth gains importance compared to relational wealth and embodied wealth; see Fig 1 for definitions of key terms) [10,11]. When faced with early-stage market integration in situ (i.e. in place) members of self-sufficient, small-scale societies cope with these changes by collectively drawing on their existing culture. This includes expectations for social hierarchy and wealth distribution, which depending on the group, can range from near-egalitarianism to extreme forms of hierarchical elitism [12]. A remaining challenge for researchers is to specify the mechanisms by which culture mediates the effects of early-stage market integration on wealth distribution and social hierarchy. This would help to predict what aspects of culture help to promote a good life for the greatest number of people in societies confronting market integration and globalization. A better understanding of culture and wealth distribution at the early stages of market integration would also help to solve the puzzle of inconsistent wealth inequality across developed and developing market economies [13]. Continued disagreement over the root causes of wealth inequalities has led to divergent policy pronouncements for their resolution. New evidence helps to narrow future directions for research and policy but many questions remain.

Two recent longitudinal and multi-sited studies challenge previous theories about the ultimate causes of disparities in social status and wealth during market integration and help to point the way forward for research on the place of culture in wealth inequality. First, Borgerhoff-Mulder and colleagues [12] demonstrated that the existing range of wealth inequality (understood broadly as including material wealth, relational wealth and embodied wealth) from one self-sufficient small-scale society to the next was sometimes as large as that between large-scale, industrialized societies. This finding decreases the likelihood that change in population scale alone is responsible for a society’s degree of wealth inequality. Instead, the authors identified two alternatives that best predicted wealth inequality 1) the nature of the livelihood strategies in use and 2) the existing social institutions and norms of resource distribution (i.e. mobility customs, land rights, property rights and political structure).

Next, Piketty and colleagues [14] marshalled decades of national-level longitudinal data in developed and developing countries to argue, among other things, that the total wealth of a market economy is not predictive of inequality. In its place, they found that extended periods of market economic growth (usually decades) predicted higher inequality until and unless growth was combined with social expectations and political action to provide for social mobility. In this regard, they report that limits on excessive accumulations of wealth (usually via interruption of cross-generational transfers of capital) were effective. A corollary to these findings is that where interventions did not take place, continued economic growth in market economies did not self-correct inequality, as long predicted by economists such as Kuznets [15].

Taken together, this evidence suggests two societal characteristics that are essential to predicting increases in wealth inequality related to market integration. First, is the nature of the livelihood strategies in use. For example, some livelihoods allow for more rapid accumulation of material wealth while others are better at promotion and maintenance of relational or embodied wealth. Second, is the emic (i.e. locally meaningful) conception of wealth, including its valued forms, patterns of distribution and role(s) as a status determinant in social hierarchy. Of great import to this study, both livelihood and forms of wealth are known to be mediated by culture, and more specifically, lifestyle [16,17]. Cultural lifestyle is a prototype, or emic consensus, of the importance of key items (including behaviors) believed to be necessary for a good life. Lifestyle prototypes invariably include items that affect decisions on what forms of livelihood to pursue and the best types of wealth to accumulate. In the context of small-scale, self-sufficient societies undergoing in situ early-stage market integration, lifestyle can also be expected to largely reflect what has long been meaningful in the traditional culture. Studying the role of culture and status determinants in such a context therefore requires theory and a set of methods capable of capturing the level of cultural group consensus in lifestyle as well as aspects of the domain that may be contested across communities and levels of market integration.

In this study, I use cultural consensus analysis based on a cognitive theory of culture to assess prototypes of belief and behavior in the domain of lifestyle across two communities at different levels of market integration. Cognitive anthropological theory takes as axiomatic that shared culture and meaning ultimately lie within the minds of individuals [16]. Though no one person can know the complete model, surveys of relatively small and diverse groups have been shown to reliably exhaust all knowledge in a cultural domain [17]. A cultural domain, like lifestyle, is something that commands people’s attention, influences their choices and engenders discussion [18]. Here, I model the prototype for what is needed to live a good life among a unique population of relatively egalitarian and self-sufficient forager-horticulturalists in Bolivian Amazonia, the Tsimane’. While extensive research has already been carried out among the Tsimane’ on an array of topics including social status, health and demographic change, little work has been done to study the emic aspects of their cultural lifestyle, its consistency in settings of varied market integration and its relationship to other status determinants [19,20].

As a group, the Tsimane’ are at the early-stages of market integration with only limited participation in the regional market economy. However, despite a generally low-level of market integration, a well-established gradient of market exposure exists across communities as proxied by access to the nearest Bolivian market town [19,20]. These characteristics make Tsimane’ society uniquely suited to a study of culture, status determinants and early-stage market integration. In order to take full advantage of this setting methodologically, I carried out the study in several iterative phases. First, I elicited the contents of the lifestyle domain from a sample of residents taken in each of two Tsimane’ communities, one more and one less market integrated. I then used this data to build emic rating exercises to estimate group consensus in lifestyle based on expertise-weighted measures of individual knowledge. The level of sharing in cultural consensus is calculated using an inverted factor analysis that estimates an Eigen vector ratio of values from loadings on the first and second factors of the analysis using respondents’ weighted knowledge scores (0–1). Next, I also regressed these knowledge scores against a set of status determinants common in market economies (age, gender, years of education and relative material wealth) to test for social incongruities. Social incongruity (forms of which are also referred to as status incongruence, status inconsistency and cultural dissonance [21–24]) describes the deleterious effects on status and health that result when two or more status determinants conflict with one another. Finally, I endeavored to capture contested aspects of lifestyle beyond consensus by assessing residual agreement in lifestyle by analyzing loadings on the second factor. Recent evidence suggests that residual agreement coefficients can be usefully analyzed to provide additional qualitative context to contested and changing domains [25–28]. In particular, these coefficients can be interpreted as indicating membership in a subgroup that diverges in one direction or another from the overall cultural consensus.

Lifestyle has been a frequent focus of research on change and social incongruity during economic transitions because of its role as both a culturally constructed and structurally imposed mediator of social status [29,30]. This can be seen in the calculus necessary for maximizing social status, which for any one individual or household in a culture, does not simply require fulfilling a static set of behavioral and material expectations [31,32]. Members must also be knowledgeable about ongoing changes and challenges to cultural expectations (including consensus around preferred types of livelihood and wealth) and capable of balancing these simultaneous demands [33]. Testing for social incongruity between lifestyle and status determinants combined with analysis of a contested domain using residual agreement analysis, can therefore help to provide a more detailed understanding of emic social status during market integration [34,35].

Many examples of social incongruity are documented in the literature [7,36–40] across a broad array of economies, cultures and geographies. A typical example is ‘*color*’ incongruity. In parts of Puerto Rico, ascribed race takes the local form of *color*, so that *color* incongruity is a conflict between an individual’s race and socioeconomic status (SES) resulting in lowered social status overall for those ascribed an undesirable *color*. This form of incongruity has been linked to an attenuation of the usually salubrious effects of higher SES for those ascribed *negro color* compared to *blanco* color (even when controlling for objective measures of skin tone based on skin reflectance measures) [40]. As illustrated by color incongruity, the nature of social incongruity also depends on the degree to which individuals have control over their status determinants. This varies because some social determinants are ascribed by others based on physical and social characteristics (e.g. race), though the effect of most status determinants depends on a mix of both agency and structural constraint (e.g. access to education versus aptitude, hard work versus opportunities for social mobility, hunting talent versus gender-based proscriptions on behavior).

If cultural lifestyle is central to the emergence and maintenance of livelihoods and patterns of wealth elsewhere tied to the worsening of inequalities in market economies, then tracing status incongruity and residual agreement among a population at the early-stages of market integration can provide unique insight into this process. In particular, it can help to uncover novel parts of the mechanisms that drive wealth inequality at the earliest stages of market integration. In all, I investigate three key research questions:

1. To what extent is there consensus and broadly shared knowledge in the domain of lifestyle among Tsimane’ respondents across levels of market integration?
2. Is there variation in Tsimane’ beliefs about lifestyle prototypes that correlate with status determinants and level of market integration?
3. Are there patterns to social incongruity and residual agreement in Tsimane’ lifestyle that indicate the influence of market integration?

Though the cross-sectional design of this study does not allow for testing of causal hypotheses, it is ideal for exploring novel questions. For example, if culture fills a critical mediating role in the emergence of excess wealth inequality during market integration (e.g. via an influence on choices of livelihood and forms of wealth and its distribution) then we would expect to find variation in the differential lifestyle prototypes of Tsimane’ subgroups living at different levels of market integration. In particular, if prototypes have shifted away from those of a mostly-egalitarian, foraging-horticulturalist lifestyle, then a likely indicator of this process would be the presence of social incongruities between new and traditional determinants of status.

### Small-scale societies, economic change and market integration

Most in situ economic transitions from self-sufficiency to cash-based market integration among small-scale societies are driven by a set of factors that have remained largely consistent across place and time since the colonial era [41]. These factors can be summarized into three areas 1) exchange of information and goods with outsiders that engender desires for acculturation and technology acquisition, 2) encroachment pressures via deception, threat or violence, from outsiders seeking land, labor and natural resources and 3) increased population size that strains territorial carrying capacity by depleting the natural resources needed for a self-sufficient lifestyle [42–45]. Together, these factors help to determine new patterns of intergenerational wealth transmission, sometimes resulting in an increasingly more unequal distribution of opportunities via existing and emergent inequalities [46]. For this reason, determining how access to opportunity is affected by market integration and its specific contextual drivers in the short- and long-term is of particular concern in developing settings. Globally, increasing levels of inequality threaten to induce political instability and imperil the well-being of many resource-insecure populations [7,14,47].

### Social incongruity

Research on social incongruity has provided novel evidence for the multidimensional nature of social status by capturing the individual consequences of simultaneously occupying conflicted social positions [7,21]. For example, Dengah [48] found that social incongruity among Brazilian Pentecostals due to conflict between religious and secular lifestyles explained more variance in individual well-being than even SES. Similarly, Sorensen et al. [38] discovered that among Yakut reindeer herders undergoing economic change in Siberia, SES in excess of material resources resulted in higher levels of stress for respondents with market- and subsistence-based lifestyles. When considered in conjunction with Gravlee and colleague’s [49] work on *color* incongruity in Puerto Rico, these studies highlight the context specificity of social incongruities and the potential for culture to mediate inequalities in a population.

The frequent occurrence of social incongruity at different societal scales and levels of wealth also represents a puzzle as to its origins and purpose in human populations. One possible explanation comes from theories of social identity, social dominance and system justification [49]. All three contend that inequalities due to determinants that are at least partially outside of an individual’s control (like social incongruity) are rooted in the maintenance of the status quo by the elite and powerful in a society. In particular, high-status individuals (and those aspiring to high status) actively police social boundaries as a way of preserving the system that empowers them. Several common social phenomena have been empirically linked to this type of social policing including dominance behaviors, ethnocentrism, homophily, ingroup bias, intergroup conflict, outgroup antipathy, resistance and self-interest [49]. The evidence supporting these theories resonates with studies on social incongruity that support its possible role in policing social boundaries for the maintenance of norms and existing hierarchies. What is not clear, is whether the role of social incongruity is the same in non-market and market economies and therefore if social incongruity has the same consequences on social status and wealth inequality in both settings [48,50,51].

## Research setting

**Fig 2:**
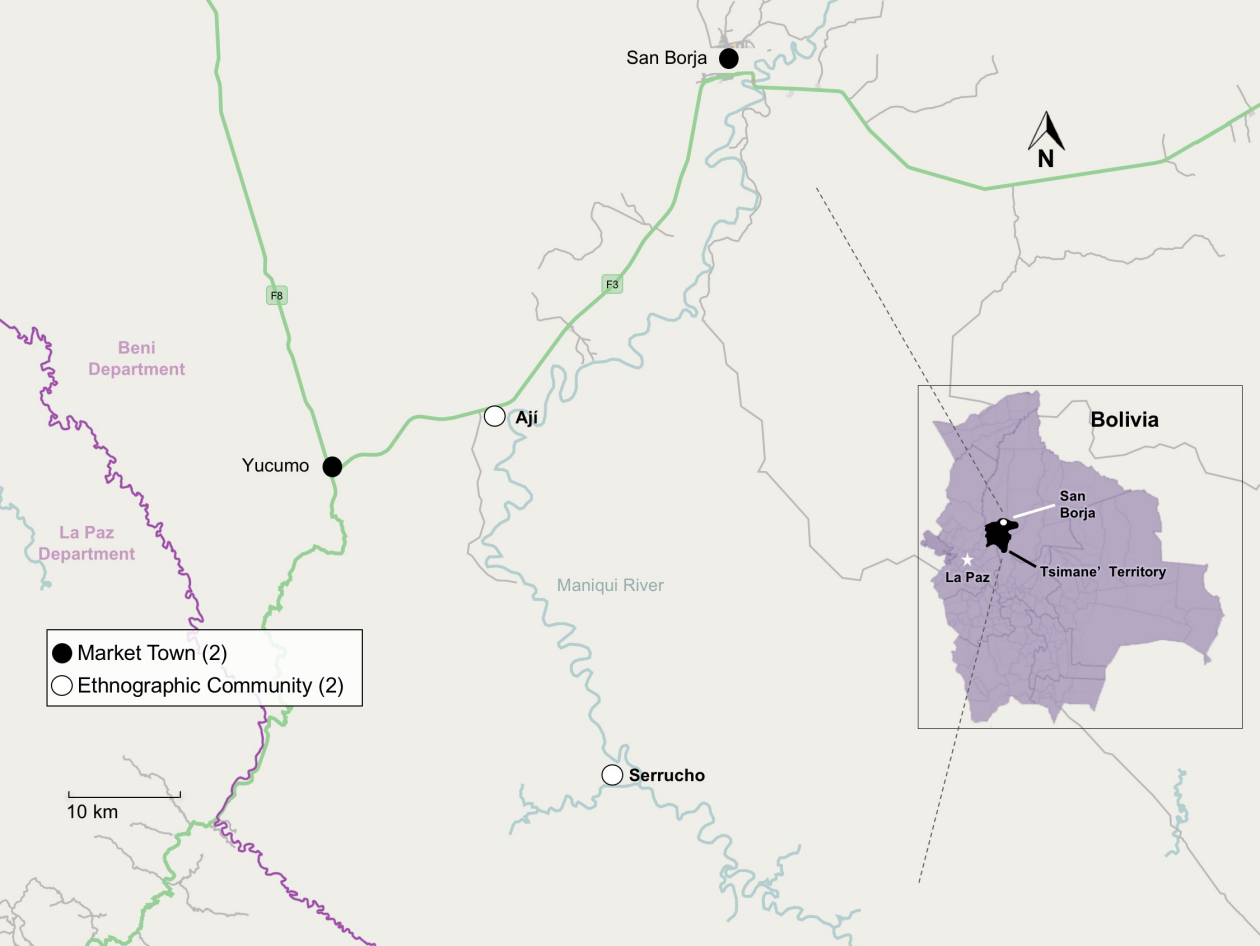
Location of the two study communities and nearest market towns.

The Tsimane’ population numbered ~16,000 in the most recent national census in 2012 [52]. Tsimane’ territory includes much of the Maniqui river and most communities lie along its banks (Fig 2). Upriver communities are typically accessible only by dugout canoe–a persistent barrier to past and current change [53]. Tsimane’ rely principally on subsistence foraging and horticulture for survival rather than wage work and a monetary economy [54]. This strategy comes with attendant risks from crop loss, over-use of natural resources and general food insecurity that are attenuated via social support and exchange networks, which helps to explain a traditional prioritization of relational wealth [35]. For residents of remote and isolated communities at the time of this research, occasional visits to market towns required lengthy trips and bore relatively high monetary costs. Furthermore, owing to their isolation and low levels of education, most Tsimane’ are monolingual in their own language, which puts them at a disadvantage in market interactions with Spanish-speaking non-Tsimane’. Despite such barriers, many Tsimane’ sign on to labor contracts (e.g. logging or weaving *cajtafa* palm leaf roof panels) when in need of a cash advance, as there are few other forms of external financial credit available [55]. A limited number of Tsimane’ communities, most of them downriver, have more moderate levels of participation in the regional market economy and fewer communication barriers with outsiders due to a higher rate of basic Spanish fluency. The gradient of market integration increases moving downriver (i.e. northeast) [20].

### Tsimane’ social status and social hierarchy

Prior to the 1970s–80s, and like many other Amerindian groups, religious belief and health maintenance were inextricably connected in Tsimane’ ontology (i.e. worldview), making the medicinal and spiritual guidance of the local *cocojsi* (shaman) powerful in the Tsimane’ social order [53]. Tsimane’ life was substantively altered when, during these years, the confluence of several significant events began to break these past ontological ties. Their symbolic climax was the loss of the last *cocojsi*, which signaled a deterioration of the strong connections between religious beliefs, traditional knowledge of healing and social order. These changes, however, were long foreshadowed by the increasing influence of Christian missionaries, western medicine, ranchers and logging companies in the area [53]. Part of what precipitated this process was a new road built through the territory–usable mostly during the dry season–that brought increasing numbers of new settlers. Unlike many nearby indigenous groups, the majority of Tsimane’ had resisted religious conversion up until this period by escaping into the forest upon confrontation or conflict with outsiders. With increasing acceptance of Evangelical and other missionaries came increased access to western education and biomedicine as well as more permanent settlements in which schools could be established [53]. Missionaries and logging companies also aided the Tsimane’ in the establishment, recognition and support of the Gran Consejo Tsimane’, which now serves as the official autonomous polity of the group in all interactions with the Bolivian federal government and with those seeking access to territorial resources.

Change in social order and exposure to outsiders brought new opportunities for political, religious, skills-based and monetary wealth status. Members of the Gran Consejo and *corregidores* (elected community magistrates) gained novel political positions, new school teachers gained novel skills and regular incomes, households selling surplus crops gained economic clout, and Christian lay pastors took up the mantel of spiritual leadership. By the mid-2000s federally funded benefits for mothers, children and the elderly became available–contingent on officially registering with the government and obtaining approved identification–further disrupting social hierarchies by introducing supplemental resources for subgroups who had previously been some of the most vulnerable and dependent [56]. Despite these changes, continuity remains in much of Tsimane’ life, including patterns of intergenerational wealth transfer and egalitarianism. For example, Hooper and colleagues [57] found that Tsimane’ parents, grandparents and siblings continue to provide significant downward transfers of food across generations but that the extent of provisioning depends on household circumstances such as productivity and the demographic composition of families. They also determined that participation in culturally meaningful social activities, like drinking *shocdye’* (beer brewed with locally available grains) among men tended to result in larger social networks (i.e. relational wealth) and increased social status.

Reyes-García and colleagues are the only previous researchers to explicitly investigate a consensus measure of lifestyle among the Tsimane’ [58,59] but their research faced a unique measurement challenge that may have affected the accuracy of results. This occurred previous to construction of lifestyle rating questions and consensus analysis, during the elicitation phase for lifestyle items. In particular, researchers asked study participants to free list components of ‘a good Tsimane’ lifestyle’ rather than ‘a good life in the community.’ What is typically the gold standard for this type of exercise (i.e. explicitly specifying the cultural group of interest), in the case of the Tsimane’ may have misled participants. This is because among the Tsimane’ the use of “Tsimane’” as a qualifier, including the common translation of *chätidye’* (a Tsimane’ word with a meaning similar to *relative* or *relation* in English), is often conflated with a description of living in an outdated way. Therefore, it is unclear if their lifestyle model documents a modern, outdated or mixed form of lifestyle [19]. Keeping this in mind, Reyes-García and colleagues reported the following five items as most important to lifestyle (in descending order): to visit kin, to spend time with close family, to have good food, to have a good agricultural plot and to be visited. Three of these items are concerned with relational wealth and two with producing embodied wealth. Among an additional 791 respondents, researchers then tested the cultural model of lifestyle against an existing dataset of behavioral measures to estimate cultural consonance. Cultural consonance measures how well a person approximates the prototype of lifestyle in their own beliefs and behaviors. It has been associated with improved health and well-being across several populations [60] but this expectation was not reflected in Reyes-García and colleague’s study. Instead, there found only mixed evidence for salubrious effects from lifestyle consonance. More research on modern Tsimane’ lifestyle is needed to verify and clarify the existing research.

### Social order and social hierarchy among Tsimane’ men

For Tsimane’ men, knowledge of new ways to make a living and higher rates of interaction with outsiders have begun to spur a livelihood transition [61]. Traditional livelihoods are now commonly supplemented by non-traditional means, the majority of which are physically demanding (e.g. farming crops for surplus, logging on contract, or wage labor like clearing jungle for ranchers) and involve substantial risks (e.g. crop-loss, physical injury, legal peril or employers who refuse to pay) [55]. As compared to women, who are the primary caretakers of children, men’s status is tightly tied to their ability to provide for their household’s needs and to support broader familial and social networks to maintain relational wealth. This has made Tsimane’ men an obvious target for investigations of social status.

Among the two principal groups of researchers who have studied social status explicitly among the Tsimane’, both have principally focused on men. The first group is led by von Rueden and the other by Reyes-García [18,62–65]. von Rueden and colleagues [18] first studied relative social status among 57 Tsimane’ men as ascribed by third-party male evaluators using four measures of perceived and functional status (i.e. respect, community influence, gets way in a group and wins dyadic fights) drawn from published theory and research. They applied their method in a relatively market-integrated Tsimane’ community, Ton’tumsi, and found that men with more traditional skills were conferred a qualitatively different kind of social status, respect, than those with non-traditional skills who instead possessed higher community influence. They also determined that social support best predicted getting one’s way in a group. These results indicate that increased community market integration may influence the kind and function of status acquired via different kinds of wealth. In another study [65], Tsimane’ communities closest to a market town were found to have higher population densities and higher levels of inequality in informal political influence between residents, suggesting a role for market integration in increasing the severity of relational wealth inequality between men. Finally, in a sample of 199 Tsimane’ men from four communities [66], higher incomes were associated with higher levels of objectively-measured stress, while peer-evaluated status was associated with less stress. This suggests that there may be trade-offs for pursing monetary and material wealth over relational wealth among Tsimane’ men. That is, in these four communities higher monetary wealth was correlated with relatively less embodied wealth (more stress), while increased relational wealth was correlated with relatively more embodied wealth (less stress).

Reyes-García and colleagues also studied social status among Tsimane’ men. They conducted a study on social rank [62] using third-party nominations of ‘important people’ in the community to estimate community social rank for 289 Tsimane’ men from the same community. Social rank was found to correlate with three separate measures of nutrition, indicating another possible link between relational wealth and embodied wealth among Tsimane’ men.

### Social status and social hierarchy among Tsimane’ women

Most of what we know about the determinants of social status and social hierarchy among Tsimane’ women is based on ethnographic accounts. For example, Tsimane’ women spend the majority of their time either working at home, taking care of children or foraging and farming near the household (often accompanied by children) [54]. Within the community, women exercise their power as sources of social information and cultural knowledge [67] but rarely hold modern political positions, such as corregidor, or occupy new high status skills-based roles such as school teacher. As is common in many societies, gender in Tsimane’ society plays a determinant role in social status including disadvantages for women reflected in high rates as reported victims of intimate partner violence and a lack of equality in opportunity for new income-generating strategies [64,68]. Common barriers to women’s market participation include social expectations that discourage work with outsiders and male peers, difficulty in taking time away from family obligations for short-term migrations, lower levels of education and fewer Spanish language skills. These barriers represent conflicts that originate from the traditional role of women in Tsimane’ society and when combined with discrimination against Tsimane’ by other ethnic groups, creates a double burden of gender- and ethnic-based inequity that limits access to, and acculturation into, Bolivian society [69,70]. These conclusions are supported by a new comparative study by von Rueden et al. [71] that found women at a disadvantage in community-level influence linked to body size, education, and social networking. The authors propose that each of these links act as status determinants for both genders but that women are at a disadvantage in acting on them. For example, the male advantage in body size is mostly pre-determined, while cultural expectations for in-home labor limits girl’s access to schooling and a limited number of prescribed social strategies are open to women that would facilitate maintaining a large social network. Expanding our knowledge of status determinants among Tsimane’ women will help to clarify the influence of economic transition on all of Tsimane’ society.

### Ethical considerations

Research design and all informed-consent and data collection procedures were approved by the Institutional Review Board 02, University of Florida (IRB-02-U-341-2011) and the autonomous authority of the Great Tsimane’ Council. Due to high rates of illiteracy among study participants, a waiver to written consent was granted for the study and verbal consent was obtained from all participants.

## Methods

This study aims to 1) model and assess level of agreement, or consensus, on the emic nature of lifestyle in two communities of relatively egalitarian Tsimane’ forager-horticulturalists at two levels of market integration, 2) evaluate associations between individuals’ cultural lifestyle knowledge and status determinants for evidence of social incongruities and 3) compare the quantitative and thematic patterning of lifestyle items and ratings between population subgroups for insight into the significance of lifestyle variation.

### Study design

Below, I present detailed descriptions of sampling and measurement methods for each of the phases of research preceded first by a brief summary of the general study design. Prior to the start of this research, I completed extensive participant observation in each of the two study communities over a period of one year [72]. The research described herein began with two iterative phases of data collection and analysis to build and test a cultural consensus model of lifestyle [73] including free listing activities and ratings exercises. The analysis of free listing results was used to develop the ratings exercises, which were then piloted and administered in a subsequent phase. Once consensus analysis of the ratings was complete, I performed two rounds of regression analyses on the loadings for the first and second factors of the lifestyle model to 1) identify possible subgroups within either factor of lifestyle and 2) assess the presence of social incongruity between aggregate subgroup preferences and status determinants (i.e. age, gender, years of education and relative material wealth). Next, to aid in interpretation of the consensus and regression results, I plotted deviation scores between subgroups’ aggregate loadings on lifestyle model items and the ‘ideal’ consensus answer key ratings as calculated in consensus analysis of the entire study sample. Lastly, using a truncated set of lifestyle items (for ease of interpretation), I assigned themes to plots of subgroup item deviation scores based on previous ethnographic data, published literature and input from Tsimane’ translators.

### Sampling and variables

Aided by two Spanish-Tsimane’ translators (Ignacio Huasna and Orlando Durbano Tayo), I collected all of the study data in two Tsimane’ communities (Fig 2) located roughly two-hours (Ají) and two-days (Serrucho) travel time, respectively, from the nearest market town. All interviews occurred during 2012–2013 and were given in the local language (Tsimane’) with a translator present to ensure accuracy and ease of communication. The two study communities were selected to complement one another based on 1) average travel time from the nearest market town as a proxy for level of market integration 2) sufficient size to facilitate the purposive life stage sampling method and 3) ethnic makeup (>95% Tsimane’ in each community) to help ensure an unbiased elicitation of the cultural lifestyle model without needing to qualify lifestyle as ‘Tsimane’ lifestyle.’ I chose travel time to the market town of San Borja as the study proxy for level of market integration, as it has proved reliable in past studies [56]. From Ají, travel one-way to San Borja on clear roads takes 50 minutes and costs $2–5 USD. For residents of Serrucho, travelers must first canoe (8–16 hours motorized, $5–10 USD) or hike (two days) to reach the highway near Ají before continuing on to San Borja.

To maximize variability in potentially important status determinants related to market integration, I used life stage purposive sampling based on age groups, gender and relative material wealth (Table 1) [74]. I limited the range of participants in the free list exercises to Tsimane’ adults aged 16–70 years in order to maximize comprehension of the activity and reliability of the responses. I expanded this range to 15–75 years for the ratings exercises, which are usually easier for participants to comprehend. I conducted free list exercises and ratings surveys with 32 and 69 adult respondents, respectively. The ratings subsamples represented 30.4% and 21.6% of the eligible populations in Ají (*n* = 115) and Serrucho (*n* = 157), respectively, based on contemporaneous community censuses [72]. Age, education, gender and ethnicity were assessed via self-report and verified against government identification cards (when available) and with community records kept by local officials. Due to highly variable incomes and commonly shared resources, I used a dichotomous measure of relative household material wealth, rather than recent income, to assess financial wherewithal. Material wealth was ascribed as more or less wealthy than average according to the mean of three third-party assessments by senior community members who ranked household wealth based on “the money this household has, the things they own and the things they can afford to buy, like food, clothing, toys, tools, and weapons.”

**Table 1:** Purposive life stage samples^a^.

	15–34 years	35–49 years	≥50 years	Female	Higher material wealth
Free list exercises (*n* = 32)	0.44	0.28	0.28	0.53	0.50
Ratings exercises (*n* = 69)	0.45	0.29	0.26	0.48	0.49

^a^Subsamples reported as proportions of sample total.

### Free list and rating exercises

With each respondent, I used the same free list elicitation, which translates as “List all of the people, items and activities a person needs to live a good life in your community.” When, on occasion, respondents did not readily name at least 20 items, I used follow-up prompts for additional ‘people,’ ‘items’ and ‘activities’ most needed for a good life. A typical free list interview lasted 20–40 minutes and I recorded all items in a notebook. Later, I combined free lists into a database and Tsimane’ translators aided in confirming and condensing similar responses into composite elements (study data is available at OPEN ICPSR http://doi.org/10.3886/E104423V3).

Of note, in this study, a pile sort activity–typically used to elicit how knowledge is structured within a cultural domain–was excluded from development of the rating exercises due to a poor level of observed comprehension among respondents during pilot exercises. This was likely due to the exclusive use of pictures to represent complex lifestyle items for piles sorts, without accompanying written descriptions–an approach necessary due to widespread illiteracy.

Ratings questions were based on 38 items (37 from the free list results and *isätri*, a charm used when hunting, drawn from participant observation). The ratings question template translates as, “How much does someone need _____ to live a good life in your community?” on a scale from 1 (“They don’t need them/those at all”) to 5 (“They need them/those very much”). Exercises included the 25 most-mentioned items (listed by >50%), six medium-frequency items (listed by 50–25%), six low-frequency items (listed by <25%) and one unmentioned but observed item, *isätri*. I chose to include *isätri* because it was frequently a topic of discussions about hunting in Serrucho, but not Ají. The inclusion of high-, medium- and low-frequency free list items in ratings exercises has been shown to improve domain measurement when there is potential for little variation in ratings across high frequency items [75]. Pilot ratings and the final results of this study bear this out, with the average rating of the 20 top-rated lifestyle items ranging between 4.5–5.0/5.0. I also used reversed ratings for half of the questions after detecting automatic acquiescent response bias during pilot ratings exercises (i.e. “yea-saying” or respondents giving all high ratings often due to boredom, predictability of answers or social desirability bias) [76]. Contrary to previous research on Tsimane’ lifestyle [59,77], I did not quality lifestyle as ‘a good Tsimane’ lifestyle’ in the final ratings exercises because during pilot tests participants often misunderstood a ‘good Tsimane’ lifestyle’ to mean “in the time of the grandparents,” as one respondent stated–an outdated way of life. Instead, I asked about “a good life in the community,” to emphasize modern cultural perspectives within communities populated by >95% self-identified Tsimane’. Ratings exercise were conducted among a fresh sample of participants separate from respondents who completed free list exercises.

### Consensus and regression analysis

I conducted consensus analysis of the ratings exercises using Analytic Technologies’ UCINET 6 software [78], which provides both formal and informal versions of consensus based on different analytic assumptions and data types. The informal model uses reliability analysis and has the advantage of allowing the use of interval and ranked response data while the formal model relies on a mathematical model. The validity of results from these two approaches has been demonstrated to vary little when analytic expectations such as number of items, congruity of content, sample size and sample diversity are met [73]. Consensus analysis is similar to a factor analysis except inverted to examine participants’ lifestyle knowledge scores, rather than individual lifestyle items. It produces four key outcomes, including 1) individual cultural knowledge scores (0–1.0), 2) model goodness-of-fit, in the form of an eigenvalue ratio between loadings on the first and second factors (>3:1 is the minimum ratio recommended for a determination of consensus), 3) a cultural consensus answer key that estimates the ‘ideal’ or prototypical rating of each item/characteristic in the model based on the combined, knowledge-weighted item ratings from all respondents and 4) residual agreement coefficients (±) which load onto the second factor of the lifestyle model and can indicate membership in a subgroup that diverges in one direction or another from the overall cultural consensus.

Following consensus analysis, I used the loadings on the first (i.e. knowledge scores) and second (i.e. residual) factors of lifestyle to test for associations with subgroups using linear regressions [25]. For each subgroup found to be associated with loadings on a factor, I then calculated item-specific subgroup deviation scores for every item. Item deviation scores are an extension of a core concept in consensus analysis: that the ratings answer key represents the ideal emic ratings for the cultural domain examined. Deviation scores build on this by allowing comparisons at the subgroup level, which is done by calculating the relative difference (±) between the consensus answer key and the average item ratings among all members of a subgroup. Based on the results of regression analyses, I calculated deviation scores for gender subgroups associated with the first factor loadings and community/market integration and wealth subgroups associated with the second factor loadings, following steps outlined by Dressler [25].

Finally, to further explore the meaning behind lifestyle heterogeneity in the second factor, I plotted subgroup deviation scores for the items that varied most as determined by natural breaks in the data (supplementary analysis and the cutoff plots used to determine item inclusion in these sets are available at OPEN ICPSR http://doi.org/10.3886/E104423V3). Briefly, one-dimensional plots of absolute item deviation scores were used to establish natural cutoffs when irregular gaps occurred in the plots. For the gender subgroups, the items within the cut-off represent the highest 15% (*n* = 5 of 38) of absolute deviations from the consensus answer key, while for community subgroups and wealth subgroups the items within the cut-offs represent the highest 35% and 30%, respectively, of lifestyle items (*n* = 11 and *n* = 13). To ensure the representativeness of these subsets for gender-, integration- and wealth-based subgroups, I reanalyzed each data set to look for substantive alterations in meaning after both adding and subtracting ~10% of total lifestyle items. This step produced no meaningful differences from the original analyses. Lastly, I analyzed and categorized the resulting plots for common themes based on insights drawn from ethnography and consulted Tsimane’ research assistants to ensure emic validity.

## Results

Respondent samples were relatively balanced in key demographic subgroups and between communities for both exercises (Tables 1 and 2). Free list results show that 20% of all items mentioned (57 of 292) were named ≥10 times (Table 3). These include the three most frequently listed items, 1) *crops*, including each reference to individual crops (e.g. rice), 2) *market supplies and tools* produced outside of the community (e.g. machetes) and 3) *purchased food* produced elsewhere (e.g. crackers and cooking oil). The highest rated items did not always match the items with the highest combined free list frequency because the process of combining similar free list items (e.g. each individual crop mentioned) while retaining original frequency counts to indicate the importance of items, inflates frequencies. Table 3 lists each lifestyle item/characteristic, its calculated ideal rating in the market lifestyle model and modified ideal ratings adjusted for subgroup averages.

**Table 2:** Descriptive statistics for ratings samples from Ají and Serrucho.

Variable	Ají (*n* = 35)	Serrucho (*n* = 34)	Total (*n* = 69)
Age (years)	37.7 (± 17.9)	43.4 (± 16.9)	40.7 (± 17.5)
Education (years)	4.5 (± 3.4)	2.4 (± 1.8)	3.4 (± 2.9)
Gender			
% women	54.5	52.8	53.6
Material wealth			
% less wealth	37.1	55.9	46.4
Per-person wealth*	3458 (± 4897)	3079 (± 3528)	3259 (± 4226)

*in BOB (1 USD = ~6.9 BOB).

Per-person wealth data is based on the value of a common basket of household goods divided by the number of household members as assessed in a separate epidemiologic survey described in Schultz [72].

**Table 3:** Lifestyle items, rating answer keys and ranks.

Item	Consensus answer key^a^	Serrucho answer key^b^	Less wealthy answer key^c^	Item market rank	Free list frequency rank^d^
Care for children	4.95	4.94	5.00	1	15
Clean water	4.94	4.97	5.00	2	18
Forest meat and fish	4.93	4.97	4.91	3	5
Crops and cropland	4.90	5.00	5.00	4	1
Family	4.90	4.81	4.97	4	23
Good food	4.89	5.00	5.00	6	17
Market supplies and tools	4.88	4.89	4.97	7	2
Livestock	4.86	4.81	4.88	8	4
Visits between family	4.85	4.81	5.00	9	8
Education	4.84	4.81	4.84	10	12
Good house	4.84	4.83	4.91	10	21
Listen to Radio Horeb news	4.81	4.72	5.00	12	26
Purchased food	4.80	4.83	4.91	13	3
Shopping trips	4.80	4.75	4.78	13	9
Good clothes	4.71	4.53	4.72	15	21
Biomedicine	4.71	4.78	4.81	15	7
Firearm hunting^†^	4.65	4.86	4.94	17	10
Weave *sáray* (cotton bags)^†^^;^ ^††^	4.59	4.83	4.94	18	6
Own electronics	4.59	4.53	4.78	18	18
Reside in birth community^†^	4.59	4.25	4.53	18	16
Listen to flute music	4.45	4.42	4.66	21	132
Drink *shocdye’* (homebrew)	4.33	4.25	4.63	22	11
Family helps one another^†^^;^ ^††^	4.04	4.36	4.44	23	13
*Qurpa* for health^†^^;^ ^††^	3.92	4.58	4.34	24	53
Forest medicine^†^^;^ ^††^	3.76	4.25	4.34	25	18
*Shịshi* pepper bushes^†^	3.63	3.00	3.81	26	23
Dogs^††^	3.45	3.39	3.88	27	23
Non-family helps	3.36	3.39	3.13	28	14
Zinc metal roof^†^^;^ ^††^	3.36	3.67	3.75	28	102
*Väij* fruit tree ritual^†^^;^ ^††^	3.03	3.69	4.03	30	59
Tsimane’ traditional dance^†^^;^ ^††^	2.97	3.50	3.59	31	132
Bow hunting^†^^;^ ^††^	2.82	3.94	3.63	32	132
Small family^†^	2.80	2.36	2.88	33	102
Gas stove	2.51	2.50	2.38	34	132
Traveling merchants	2.30	2.22	2.19	35	83
*Isätri* for luck in the hunt^†^^;^ ^††^	2.15	3.03	2.69	36	.
Homegrown *côs* (tobacco)	1.86	2.03	1.94	37	60
Non-Tsimane' friends^††^	1.80	1.92	1.47	38	132
Standard Deviation	1.0	.94	1.0		

^a^Consensus ratings range from 1–5, with five being the most important. The consensus key was calculated using the matrix of all individual item ratings weighted by respondent knowledge scores.

^b^Adjusted for average ratings deviations from the consensus key among Serrucho residents.

^c^Adjusted for average ratings deviations from the consensus key among respondents with less wealth.

^d^Based on free list frequencies, with items of the same frequency assigned the same rank.

(.) Item not mentioned in a free list but observed during ethnography

^†^Items with largest deviations among Serrucho residents

^††^Items with largest deviations among respondents with less wealth.

### Market integration and cultural lifestyle

The consensus analysis of lifestyle items demonstrates a level of sharing within the first factor sufficient to conclude moderate consensus (Table 4; Eigenvalue ratio 4:1) [79]. The first factor accounts for 72.7% of sample variance and the average knowledge of raters was 0.63 (± 0.12). Recommended cutoffs for the standard deviation statistic produced by consensus analysis using the informal model have not been published. However, the deviation statistic did meet the cutoff for a formal analysis model, (≤0.14 for this sample size and number of questions) as proposed by Hruschka and Maupin [80–83]. Table 4 lists consensus results in each community and combined.

**Table 4:** Cultural consensus in the first factor (‘market lifestyle’) in Aji, Serrucho and combined.

	Ají	Serrucho	Combined
Ratio of first to second eigenvalue	7.26	4.4	4.0
Mean cultural knowledge (± *SD*)	0.67 (± 0.11)	0.64 (± 0.16)	0.63 (± 0.12)
Range of cultural knowledge	0.25 to 0.82	0.1 to 0.83	0.28 to 0.79

**Fig 3:**
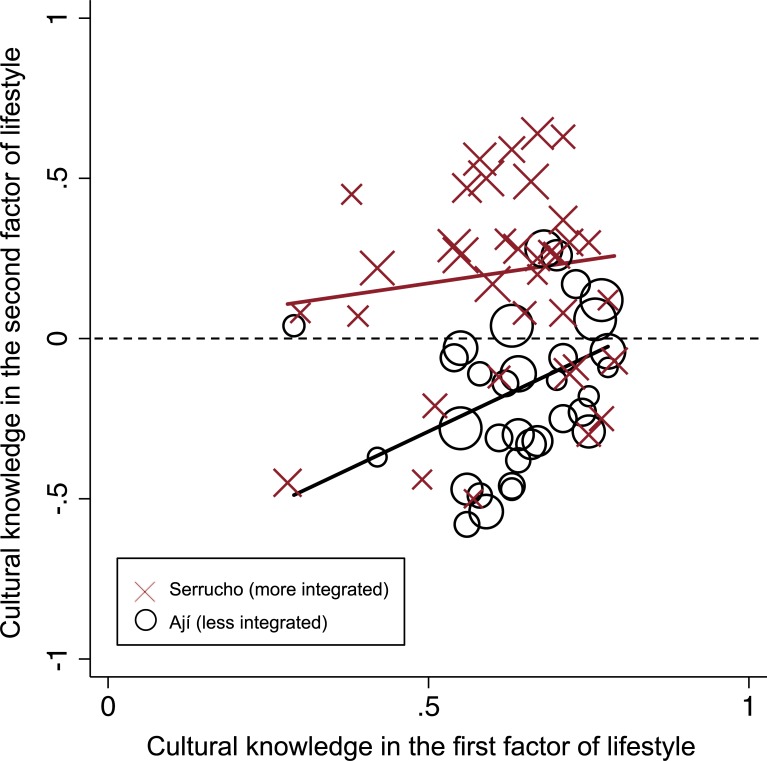
Scatterplot of lifestyle knowledge (factor 1) and lifestyle (factor 2). Markers sized by age; community trend lines match label colors; dotted line **indicates zero on the x-axis**.

In answer to the first research question, I find that Tsimane’ respondents maintained consensus in a cultural model of lifestyle despite the presence of some heterogeneity. Based on the model contents and ratings for the first factor, as well as the results of residual analyses described below, I label the cultural consensus model with its generally higher ratings for market-based items, *market lifestyle*. By contrast, I label residual agreement in the second factor *syncretic lifestyle* because of the nature of its comparative ratings, which are closer to parity for market-based and traditional items implying more of an amalgamation of lifestyles. The finding of subcultural heterogeneity is consistent with many published results from consensus analyses that regularly include residual agreement in the second factor, especially in contested or changing cultural domains [25]. Fig 3 is a plot of the loadings on the first and second factors of lifestyle and shows a clear demarcation between respondents from the study communities along the y-axis.

### Lifestyle variation across subgroups and market integration

**Fig 4:**
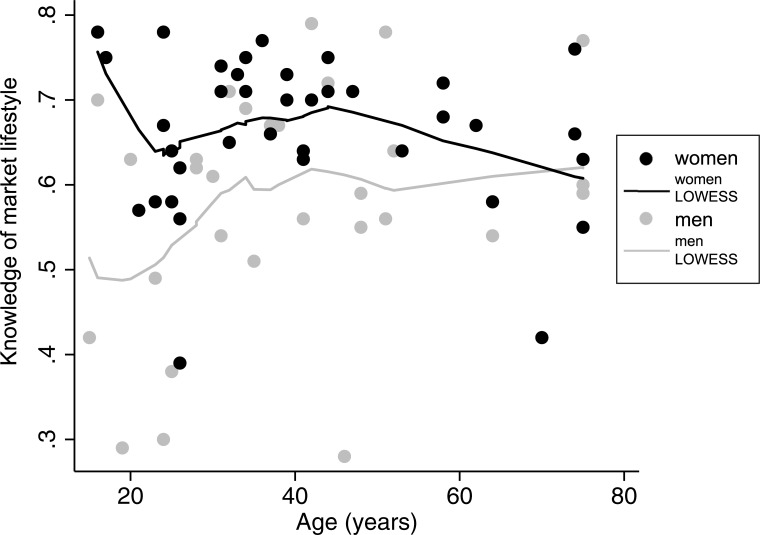
Nonparametric LOWESS of lifestyle knowledge by respondent gender and age.

**Fig 5:**
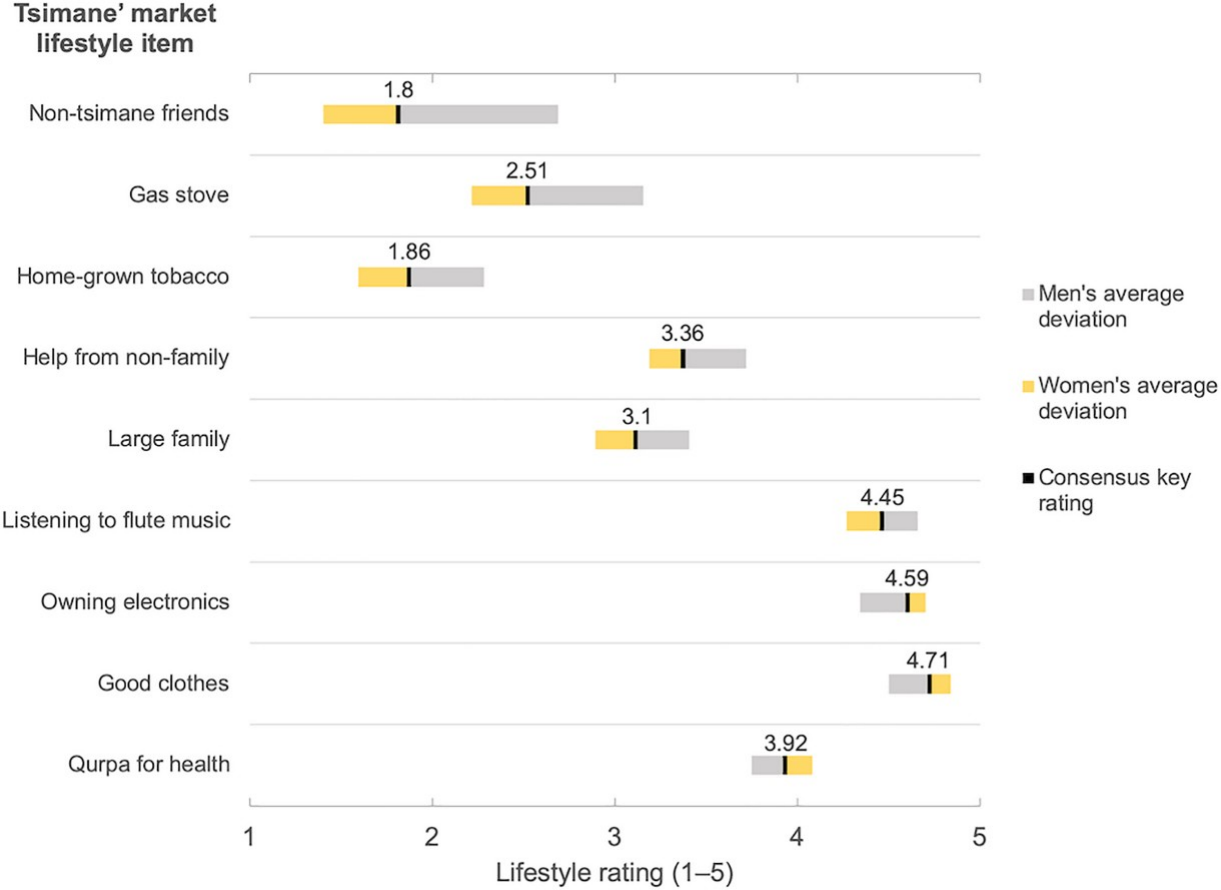
Largest ratings deviations (upper quartile) from market lifestyle by gender.

Table 5 displays the results of regression analyses of market lifestyle and syncretic lifestyle on explanatory variables. In response to the second research question, I find that women (β = .342) tend to be more knowledgeable of the market lifestyle prototype than men while no other explanatory variables are even weakly associated. Fig 4 demonstrates this difference among respondents in a non-parametric LOWESS (Locally Weighted Scatterplot Smoothing) of market lifestyle knowledge against age and stratified by gender. There is a higher level of lifestyle knowledge among women across stages of the life course and the plot also suggests that younger women have the keenest knowledge of market lifestyle, though this is not supported by the regression results. Fig 5 includes the top quartile of items for which market lifestyle ratings of men and women most diverged. Men varied most in their higher ratings for items that contribute to relational wealth, including non-Tsimane’ friends, large family size, non-family helps, social tobacco smoking, and gathering to listen to flute music. The only exception to this pattern was men’s higher ratings for gas stoves, an emically conspicuous material item that is rarely used in communities since there are no accessible sources for the fuel needed to use them. In comparison to men, women’s rating averages only strayed substantially from the market lifestyle prototype with lower ratings for having non-Tsimane’ friends. Other deviations were much smaller.I also find that more relative material wealth (β = –.331) and residence in the less market integrated community of Serrucho (β = .456) are associated with a syncretic lifestyle, supporting the existence of lifestyle subgroups. Education and age did not correlate with loadings on either lifestyle factor.

**Table 5:** Regression of market lifestyle and syncretic lifestyle coefficients on explanatory variables (standardized regression coefficients).

	Market lifestyle	Syncretic lifestyle
Age	.146	.151
Female	.342***	.016
Education	.160	–.111
Wealthy	–.127	–.331***
Less integrated	–.136	.456***
*R*^*2*^	.152*	.501***

Note

*p < .1

**p < .05.

***p < .01.

### Market lifestyle

Among the top-rated items in Tsimane’ market lifestyle (>4 on a scale of 1–5), over 40% were explicitly market-related (e.g. manufactured items and tools, market-acquired livestock or education). The remaining top-rated items consisted of high-quality versions of basic necessities that are at least sometimes market-related (e.g. *good* clothes, a *good* house and *good* food) and various kinds of relational wealth (e.g. care for children, having family around and help from family). Taken together, the contribution of market items to the lifestyle model appears substantial while still overlapping with some traditional approaches to fulfilling basic necessities and maintaining social customs. When traditional and non-traditional market alternatives both appeared, market items were typically rated higher with the exception of some food items. For example, electronics for entertainment were valued more highly than Tsimane’ flute music or dancing, hunting with a firearm was valued over bow hunting (and over the use of Tsimane’ *isätri* charms for success in hunting), biomedicine was valued over forest medicine or alternative treatment modalities like the qurpa (see below) and education was valued over living in one’s own village.

#### Syncretic lifestyle and level of market integration

**Fig 6:**
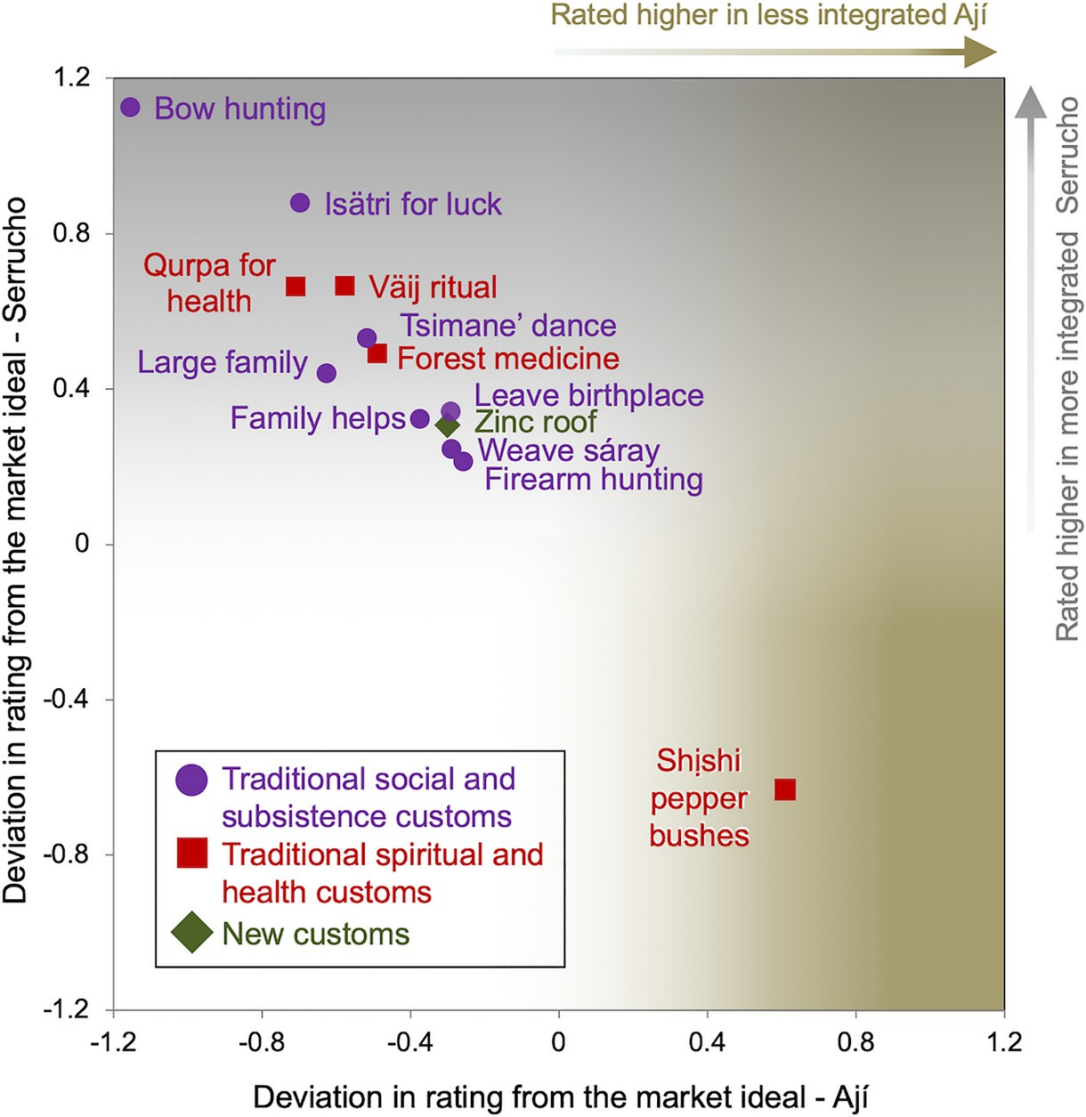
Items and characteristics that deviate most (±) from the market lifestyle prototype among respondents at two levels of market integration. Higher positive deviation scores on the Y-axis and within the grey gradient indicate higher ratings from respondents in less integrated Serrucho. Higher positive deviation scores on the X-axis and within the brown gradient indicate higher ratings from respondents in more integrated Ají.

Results of residual agreement analysis indicate a more syncretic lifestyle is preferred among respondents from the less market-integrated community of Serrucho. This result complicates interpretation of associations between market integration and lifestyle, since overall both communities agree on a market lifestyle model, but respondents in Serrucho also maintain a set of more traditionalist preferences. Fig 6 plots directional deviation scores (±) for a truncated set of the most divergent items in this subgroup. Twelve of 13 items are rated higher in less-integrated Serrucho while just one is rated higher in Ají. Table 2 also denotes (^†^) each of these 13 items along with their ideal market lifestyle rating and adjusted syncretic rating based on subgroup deviation scores. Below, I organize these 13 items into themes and contextualize each according to respondents’ descriptions from the free listing exercises.

First is the category of *traditional social and subsistence customs* valued primarily for their utility and symbolic power as part of a self-sufficient, small-scale forager-farming lifestyle. This set of eight items was exclusively preferred in less-integrated Serrucho: 1) handmaking and hunting with bows and arrows, a socially-acquired skill that requires extensive training and practice, 2) residing outside of one’s birth village, in order to establish new, or maintain old, social ties with blood relatives and new affiliative family, 3) use of a subcutaneous *isätri* pebble charm, believed to increase success in the hunt by providing a supernatural advantage, 4) gathering to do ritual Tsimane’ dances, which are believed to build good will among the community, 5) avoiding a small family to demonstrate success, fortify social ties and support a subsistence lifestyle, 6) exchanging help between family members, to support a subsistence lifestyle and accrue social capital/relational wealth, 7) weaving traditional cotton carrying bags, or *sáray*, used to transport everything from crops to small children for foraging and field work, 8) having a firearm to hunt game, which are still easily hunted in more remote and isolated Tsimane’ communities like Serrucho.

Second, four items that deviate from market prototypes based on level of community market integration can be categorized as *traditional spiritual or health customs*. That is, each is believed to be protective or curative of the spirit and/or health thereby producing embodied wealth. Three of four items in this category were preferred in the less market-integrated community of Serrucho 1) practicing the annual *Väij* fruit tree ritual (peach palm, the tree used for bow making and to ferment a seasonal type of homebrew, *buri*), among household members at first fruiting to protect from natural and supernatural mishaps and reinforce relational wealth, 2) a *qurpa* (an alum/double sulfate salt), acquired through trade with Aymaran highlanders for divining and curing common illnesses such as cough and fever [84] and 3) use and knowledge of locally-foraged forest medicine, which includes common items such as certain types of tree bark that are ingested to relieve pain and decrease inflammation. The fourth item, planting a *shịshi* red chili pepper bush outside one’s home, was preferred in more-integrated Ají. *Shịshi* and their bright red color are thought to offer powerful protection to families from certain kinds of sent sickness and other natural and supernatural dangers. Their exclusive preference in Ají is notable since, on average, residents rated traditional items lower there but prioritized this item to promote embodied wealth.

Finally, the third category is *new customs*, which indicates items that are novel for the Tsimane’. In this case, having a zinc-plated aluminum roof is a new custom preferred in Serrucho where roofs are traditionally woven from harvested palm leaves. *These cajtafa* roof panels, however, are no longer a practical choice for personal use in Serrucho after a decade of the community serving as a hub for commercial buyers of roof panels has made *cajtafa* scarce locally.

#### Syncretic lifestyle and material wealth

**Fig 7:**
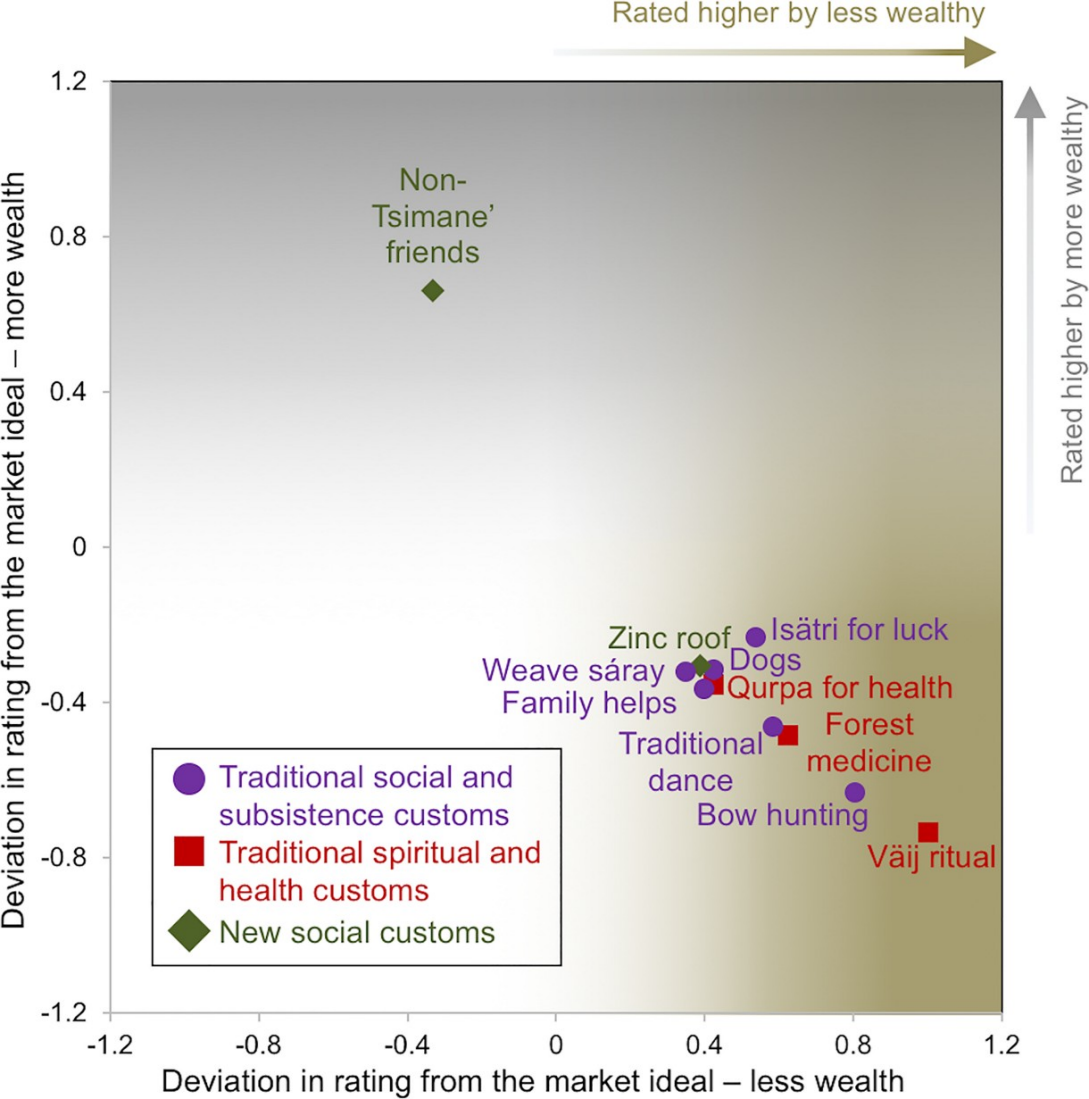
Items and characteristics that deviate most (±) from the market lifestyle among respondents in dichotomized wealth groups. Higher positive deviation scores on the Y-axis and within the grey gradient indicate higher ratings from those with more wealth. Higher positive numbers on the X-axis and within the brown gradient indicate higher ratings from those with less wealth.

To better understand variation in subcultural preferences for a syncretic lifestyle by relative household material wealth Fig 7 plots the 11 items within the cut-off for the largest absolute deviation scores from the market lifestyle prototype. Ten of 11 items are favored by participants with less wealth, while just ‘non-Tsimane’ friends’ is favored by participants with more wealth. Table 2 also denotes (^††^) these 11 items alongside their consensus answer key ratings and adjusted syncretic answer key rating based on subgroup deviation scores. Below, I organize the items into three themes and contextualize each based on respondents’ explanations from the free listing exercises.

First, is the category of *traditional social and subsistence customs* (see description above), which includes six items that were rated more highly by respondents with less material wealth including, 1) hunting with handmade bows and arrows, 2) gathering to do ritual Tsimane’ dances, 3) use of an *isätri* pebble to bring success in the hunt, 4) having dogs that help deter thieves from the homestead and aid in hunting, 5) exchanging help between family members and 6) weaving *sáray* carrying bags. The second category is *traditional spiritual and health customs*. All three of the items in this category were rated higher than the market prototype among respondents with less wealth including 1) practicing the seasonal *Väij* fruit tree ritual, 2) use and knowledge of forest medicine and 3) *qurpas* to aid health. Finally, the third category *new social customs* describes lifestyle characteristics respondents consider helpful in their attempts to increase household income or find work outside of the community. *Non-Tsimane’ friends* is the only item in this category and it was rated more highly by respondents with greater wealth who considered these connections a form of relational wealth that supplies the information needed to find work and increase material wealth.

In answer to the third research question, I find that the patterning of lifestyle by gender (differences in market lifestyle) and material wealth (residual agreement in syncretic lifestyle) indicates that the domain is contested due to conflicts between traditional priorities and market priorities. I also find that the patterning of syncretic lifestyle by level of market integration is similar to the patterning of lifestyle by subgroups of more- and less-wealthy respondents, including the two central themes in the analysis of deviations, *traditional social and subsistence customs* and *traditional spiritual and health customs*. Taken together, this evidence supports the tentative conclusion that market integration has differentially impacted the modern Tsimane’ prototype of lifestyle. The first factor of lifestyle, in particular, explains the vast majority of variance in participant lifestyle knowledge and can be understood to largely favor market alternatives. That said, gender was the only status determinant associated with this factor of lifestyle knowledge. For the second factor of lifestyle, both relative wealth and market integration were associated with factor loadings but overall these loadings accounted for less than a fifth of the total consensus analysis model variance. Future research is needed to determine definitively whether the conflict between men and women regarding lifestyle knowledge, or the existence of wealth- and integration-based subgroups indicated by residual agreement analysis, represent social incongruities that can help to predict wealth inequality and well-being among the Tsimane’.

## Discussion

The study findings build on and deepen our knowledge of the role of culture in early-stage market integration among the Tsimane’. Foremost, the results provide a wealth of information on the modern prototype of what respondents deemed necessary for a good life, including a consensus (or ‘ideal’) answer key produced with consensus analysis that can be used to compare the relative necessity of each item in the model. These comparative ratings are noteworthy because of respondents’ preferences for market-oriented items and behaviors over traditional alternatives. In addition to consensus in the market model, there is also underlying heterogeneity in loadings on the first factor of respondent knowledge by gender and on the second factor by level of market integration and relative material wealth. Given the rapid economic change ongoing within most Tsimane’ communities and the growing regional market economy with which many Tsimane’ now regularly interact, these results help to capture a modern snapshot of emic Tsimane’ lifestyle which can aid in interpretation of those changes.

In the proceeding section, I consider the implications of the study results for published literature on the Tsimane’ and broader research on market integration and wealth inequality. Specifically, I discuss five ways in which the study builds on and extends existing work, including (1) a novel study of Tsimane’ cultural lifestyle including a consensus lifestyle model that varies meaningfully from other published models of Tsimane’ lifestyle. In particular, it identifies a prevailing preference for a market-oriented lifestyle not previously measured in this forager-horticulturalist group. Past studies described a lifestyle less affected by outside influence. (2) An investigation of social incongruity between status determinants and loadings on the first factor of knowledge about lifestyle in a diverse sample of Tsimane’ adults. These analyses revealed a gendered conflict in lifestyle ratings for items representing relational wealth, which is novel for two reasons. First, because little research has been done on social status and lifestyle among Tsimane’ women compared to men. And second, because in other domains that have been investigated, Tsimane’ women tend to have better retention of traditional knowledge (e.g. identification and use of medicinal plants) but worse acquisition of market-related skills (e.g. years of education and Spanish language) [85–87]. (3) Residual analyses that confirm a subcultural syncretic lifestyle model in the loadings on the second factor of lifestyle that helps to pinpoint which groups disagree on consensus lifestyle items. The only previous published research on Tsimane’ lifestyle modeled just one dimension of the domain using elicitations that proved ambiguous when piloted in this study. (4) an in-depth qualitative and comparative analysis of the patterning of deviation scores by wealth- and integration-based subgroups. This effort helps to capture the cultural nuance of variation in subgroup lifestyle ratings, a type of analysis that has not previously been reported among the Tsimane’. It reveals that just two themes encompass almost all deviations in item ratings for subgroups that prefer syncretic versus market lifestyle. These include *traditional social and subsistence customs* and *traditional spiritual and health customs*. This result also provides an estimate of the range of variation in lifestyle ideals among the Tsimane’, which could serve as a guide for future testing of hypotheses to determine the extent of economic change in this group. And, (5) the results document variation between subgroups for lifestyle items that measure aspects of livelihood choice and preferences in wealth type and distribution. These factors are elsewhere known to be determinant in the development of wealth inequality across a broad variety of societies and can help frame future research on emic aspects of inequality among the Tsimane’. Below, I consider these five areas in detail.

### A market lifestyle among the Tsimane’

The cultural lifestyle model I describe in the results is a mix of traditional and market components with clear preferences for non-traditional market items in most circumstances where a market alternative exists. Comparison of prototypical ratings for items in the market lifestyle model and adjusted ratings for the syncretic subcultural model (see Table 3) help to demonstrate this difference. For example, in preferences for firearm hunting versus bow hunting, biomedicine versus forest medicine or market supplies/tools versus weaving *sáray* cotton bags. Among less market-integrated residents of Serrucho, who were more likely to adhere to the syncretic model than those from Ají, the average rating for use of the *qurpa* in healing was 0.66 higher (4.58/5.0) when adjusted for ratings deviations, fully 96% of the score for the use of biomedicine (4.78). Also, the difference in ratings between modern firearm hunting and traditional bow hunting is smaller among respondents from Serrucho (bow hunting is rated 3.94 and firearm hunting is rated 4.86) while ratings for all hunting are higher compared to the market lifestyle model. Only a few market lifestyle items rated in the final 38-item modelling did not have a traditional alternative. The existence of so many market alternatives is itself noteworthy as a possible indicator of change that has already occurred and supports the use of lifestyle as appropriate for investigating market influence among the Tsimane’.

The market lifestyle model reported builds on work Reyes-García and colleague’s published on Tsimane’ lifestyle [58,59] that elicited items for a ‘good Tsimane’ lifestyle’ to assess consensus in the domain. In comparing the top ten highest rated lifestyle items between this study and that, there are clear differences in market and traditional items. Here, more market-related items are in the top-ten including livestock, market supplies/tools and education, while the Reyes-García et al. model contains just one market item, ‘to acquire commercial goods.’ There are a couple of ways to interpret this difference. First, it could mean that the Reyes-García et al. study reflects a different and less market-integrated period of time (or sample of respondents). Depending on the speed of change, this is possible. Or, it could be the result of a measurement difference either due to qualitative choices made to include/exclude lifestyle ratings for elicitation exercises or, as described above, from bias introduced during elicitation of lifestyle items that altered some participant responses from modern to traditional lifestyle.

### Social incongruity in gender and knowledge of market lifestyle

On average, women had 13% more accurate knowledge of market lifestyle prototypes compared to men. To explore heterogeneity in market lifestyle by gender, Fig 5 charts the relative deviation scores for the nine items that varied most between men and women. These deviations are principally driven by men’s higher ratings for relational wealth items including the social use of homegrown tobacco, social gatherings based on listening to traditional flute music and items concerned with the maintenance of social ties to family, non-family and outsiders [88,89]. The results reflect a similar pattern to von Rueden and colleague’s findings on relational wealth among Tsimane’ men in which prosociality proved important because it tended to result in more social support in the community [18]. Independently, they also found that acculturation predicted social support in the community, a potential motivator for men’s higher ratings here for items related to relational wealth. The findings may also be indicative of a social incongruity between gender and relational wealth. In fact, other researchers have already documented the asymmetric costs to women of gendered social hierarchy in Tsimane’ communities [90]. In this case, the social incongruity between gender and lifestyle items related to relational wealth might pose a threat to women’s future social mobility. In particular, the devaluing of market-related kinds of relational wealth during the early-stages of market integration, could exacerbate existing disparities in women’s opportunities to participate in the market economy on top of existing barriers to education access, travel outside of the community and language skills. During the ethnographic phase prior to the current study, participants also reported that strict gender roles were being policed via the belief systems proselytized by Evangelical missionaries in the area. More research is needed to explain why women in this study displayed more knowledge of market-oriented lifestyle, whether it is related to a desire for greater autonomy or social mobility and what types of behaviors best predict their desired outcomes [91]

An important caveat to the interpretation of these gendered study results is that knowledge is not the same as achievement, and achievement of lifestyle was not measured explicitly in this study. Achievement of lifestyle was measured, however, in the two studies by Reyes-García and colleagues mentioned above. In their studies, only two small gendered differences were found in consonance measures of lifestyle, with associations between more consonance in men and larger body size, and more consonance in men and higher body mass index [59]. Both of these associations are indicative of a relationship between the capacity to adhere to cultural lifestyle and embodied wealth. However, depending on the local interpretation of Reyes-García et al.’s elicitation, these results may reflect modest embodied wealth benefits for consonance with a modern, mixed, or traditional form of Tsimane’ lifestyle.

Future research should test how the knowledge advantage among women detected in this study compares to their achievement, or consonance, in lifestyle. Adding to our knowledge of lifestyle incongruities among Tsimane’ women will help to clarify the influence of economic transition on the whole of Tsimane’ society, eliminate gender as a possible source of omitted variable bias in assessments of social status and help determine whether gendered incongruities drive wealth inequalities.

### The patterning of syncretic lifestyle

The multilevel and contextual variability of most social determinants makes accurate measures of social status and social incongruity particularly challenging to capture in diverse populations. For example, in Gravlee and colleague’s research [33], *color* incongruity was found to only affect participants who met the local racial criteria for being *negro* in Puerto Rico, a circumstance largely beyond personal control, yet personally and contextually specific to the setting. Similarly, Sorensen et al.’s research on the Yakut in Siberia [38] found that only those who lacked material wealth but strived for higher SES suffered from high rates of psychosocial stress. In this study, while health outcomes were not evaluated, it is apparent that respondents from the less market integrated community, Serrucho, and respondents with less material wealth, preferred a syncretic lifestyle with a more balanced set of traditional and market priorities. Further evidence of this variation is apparent in Table 4, which displays results for intra-community consensus analyses of market lifestyle. Compared to the aggregate lifestyle consensus these intra-community models show much higher agreement for market lifestyle in Ají compared to Serrucho, a possible effect of higher market exposure in Ají.

Still, identifying possible motives for market integration is difficult. For example, wealth among the Tsimane’ has been estimated to exceed that of non-Tsimane’ residents of rural lowland Bolivia when non-monetary wealth is included in estimates [92]. Combined with resource distribution expectations that result in significant downward transfers of food across generations in families [57], the evidence implies a generally high quality of life that is not likely to be a strong driver of change in livelihood practices [41]. Instead, what is more likely is that many households have been enticed into change little-by-little via exposure to modern time- and effort-saving technologies that complement traditional livelihood practices and patterns of wealth distribution in situ. An example of this process is the rapid adoption of peke-pekes. These efficient and affordable outboard canoe motors (documented here as part of the market lifestyle item ‘market supplies and tools’) have been rapidly transforming river transportation in Tsimane’ territory during the last decade. In combination with chainsaws and other modern tools, they have also greatly reduced the effort it takes for a household to expand from subsistence farming into surplus farming for profit since they facilitate easy access to far-off fields. The adoption of peke-pekes, then, represents an elective transformation with mixed motivations but whose collective effects may go well beyond immediate household goals of increasing monetary income and material wealth.

Within this context of change during the last two decades, the Tsimane’ territorial population has been increasing rapidly, mostly due to a combination of improved childhood survival rates and continued low rates of out-migration despite tightly-bounded land on which to settle [93]. Together, these patterns threaten to create a novel strain on Tsimane’ household resources, but especially among those who wish to maintain a semblance of a traditional subsistence livelihood while facing increasing difficulties in securing the space and cropland needed. It is within this context of overlapping changes that the subcultural model of syncretic lifestyle must be understood. While smaller in its overall explanatory power compared to market lifestyle (18.2% model variation explained versus 72.7%), it represents systematic variation in the lifestyle domain that parallels traditional Tsimane’ beliefs. In this way, it can help to pinpoint divisions between subgroups at different levels of market integration and those with relatively more and less wealth. Here, I explore the meaning of this patterning in light of the published research on Tsimane’ social hierarchy and social status.

#### Traditional social and subsistence customs

As outlined in the results, *traditional social and subsistence customs* make up one of two distinguishing categories of lifestyle items that deviate from the market model among subgroups. This heterodox pattern resonates with past research from Gurven, von Rueden and colleagues [18,63] who found that for Tsimane’ men traditional skills like hunting were key to maintaining respect among peers even in places where market integration has made non-traditional skills increasingly important for achieving community influence. The achievement of lifestyle under such circumstances therefore requires balancing new market priorities against traditional priorities. For example, striving to be a successful Tsimane’ hunter requires investing the time needed to acquire hunting skills while learning Spanish requires a long-term and location-bound commitment to the formal study of language. These demands can create a zero-sum struggle for the time and resources needed for each, forcing a choice between the two or reducing the quality of each. If these emerging trade-offs are combined with existing barriers such as the remote isolation of some communities or gender stratification (Tsimane’ women are not encouraged to become skilled hunters or prohibited from building certain social networks), there is a threat of social incongruity emerging for already-vulnerable subgroups [70].

#### Traditional spiritual and health customs

Integration into Bolivian society has led to important improvements in health for many Tsimane’, especially among those living closest to a market town [94,95]. However, a marked disparity in mortality experience remains for some of those in the most remote and isolated communities and this may be what is reflected in higher ratings of traditional items tied to embodied wealth [94]. Traditionally, Tsimane’ ontology mixed maintenance of health and healing with religious/cosmological belief and practice [53,96]. A change in these traditional worldviews that blend both physical and spiritual health might help to explain the deviations in item ratings in syncretic lifestyle related to embodied wealth. Two items in the syncretic model highlight the potential importance of emic views to embodied wealth and spirituality. First, is the *väij* tree ritual of cleansing done collectively by households during the first seasonal fruiting to show thanks to forest spirits, prevent illness by displaying respect for forest resources and to ensure success going forward [53]. Adhering to this ritual is believed by some to be necessary for good health. Second, is the *qurpa*, which is used in addition to, or in place of, biomedicine for both diagnosis and treatment of ailments guided by spiritual belief. While often used only for mild illness, such as a cold or flu, ethnographic informants also reported that it is helpful in treatment of sent sickness (illness believed to be sent by enemies often via third-parties who possess magico-religious powers) and forest sicknesses (believed to be acquired after disrespecting the forest in some way) that do not have standard biomedical treatments.

Since the loss of the last shaman, the retention and maintenance of traditional knowledge about health/healing and cosmological belief has deteriorated significantly [29,30]. Given the results of this study regarding embodied health, social incongruity between market-based material wealth and forms of lifestyle might be expected to continue to act as a source of vulnerability (and perceived vulnerability) for less wealthy and less-integrated Tsimane’ going forward. This is in line with research on market integration and the health experiences of other Amerindian groups, which has documented dramatic changes to social order, including higher levels of wealth inequality and worse overall health during integration into market economies [97].

### Traditional livelihood and wealth preferences

The study findings help to advance our understanding of livelihoods and wealth at the early-stages of market integration. Specifically, the market priorities of modern Tsimane’ lifestyle indicate a departure among study participants from a traditional lifestyle that favored livelihoods which were more focused on relational and embodied wealth than material wealth. This conclusion is based on the residual agreement analyses above that demonstrate a minority preference for a more traditionally-oriented lifestyle of subsistence livelihoods and relational forms of wealth. It is also reflected in the gendered social incongruity between market lifestyle and knowledge of specific relational wealth items. The devaluing of relational wealth among women in a seeming trade-off between relational wealth and material or embodied wealth, may signal increasing wealth inequalities between Tsimane’ men and women. Future research should test this hypothesis with longitudinal data.

### Limitations

The research described has several limitations. First, rigorous hypothesis testing of change requires a longitudinal design such as a cohort or panel study. In lieu of such a design, cross-sectional results must be interpreted with caution as they may reflect unanticipated confounds and cannot determine cause and effect owing to a lack of multiple measurements. Additionally, although the sample sizes I use for the chosen research design meet agreed upon standards, they are limited in geographic- and population-scope having been collected in just two communities. Another specific concern is the borderline significance (p < 0.056) for the regression model that tested loadings on the first factor of the lifestyle model, likely due to a small sample size. Also of note, is the limited number of variables (five) tested to examine status determinants against lifestyle. Combined with the limited number of highly-rated domain items included in consensus analysis (25) due to the limitations of the method, the consensus analysis may have suffered from some amount of omitted variable bias. Finally, as discussed in the methods section, I was not able to use respondent pile sorts to help organize lifestyle items into emic themes due to high illiteracy rates. I instead based themes on insights from months of ethnographic participant observation. Such post-hoc categorization of consensus data runs the risk of overlooking meaningful distinctions due to gaps in the ethnography. To minimize this risk, I also reviewed and modified themes with long-time Tsimane’ research assistants to confirm their emic face validity.

## Conclusion

In light of the results presented above and recent advances in the literature on market economies and inequality, here I draw a few conclusions on lifestyle, social incongruity and potential drivers of wealth inequality at the early stages of market integration. First, it bears repeating that the modern Tsimane’ form of lifestyle modeled in this study reflects what respondents prioritized, and aspired to, in their lives. It is not a measure of their practices but of existing cultural guidance, which may or may not be equally achievable by all Tsimane’. As a consequence, the findings presented must be understood as a part of the cultural landscape rather than its complete or static form. None-the-less, shared knowledge is undoubtedly influenced, and is influenced by, behavior and belief enacted as a part of cultural lifestyle.

What is clear as a principal result of this study is that Tsimane’ lifestyle is dynamic, not static, and appears to reflect many of the changes that have occurred within this small-scale society over the last 50 years. This includes reduced seasonal movements of communities, increasingly formalized organizational structures, more frequent exposure to outsiders of all kinds (including researchers), increased levels of wage labor, exposure to periods of intense natural resource exploitation, increased access to market goods, increased access to western education, and more influence from missionaries, to name a few [98]. The nature and extent of these changes appear to have affected core ontological beliefs about the purpose of life, the importance of social connections, the goals of livelihood, the definition of health and health maintenance and the most desirable forms of wealth, most of which are reflected in the duality of traditional lifestyle items being present alongside new market alternatives in the Tsimane’ model of lifestyle.

Modern Tsimane’ lifestyle, as described above, makes it clear that the formerly ubiquitous forager-horticulturalist livelihood that prioritized relational and embodied health for all Tsimane’, is now only one of a growing variety of livelihood and wealth aspirations. For example, many of the items within market lifestyle depart from self-sufficiency, instead placing a higher premium on monetary income and the accumulation of material wealth. This includes raising livestock, pursuing income generating work to purchase material items and acquiring education to improve employment prospects. These changes to lifestyle also include desires for novel types of wealth and wealth distribution. The novelty of these preferences is made apparent via the results of residual agreement analyses. While only 18.2% of variance was explained by the syncretic model of lifestyle, the primary consensus model of market lifestyle can only be understood in relation to the syncretic subcultural model. As a likely remnant of recent shifts in lifestyle consensus, the syncretic model is informative beyond its variance. That is, it provides a glimpse of what alternate lifestyle models may have looked like in Tsimane’ territory when market integration and material wealth had not yet achieved their current status as necessities in a good life.

Another principal finding of the study is the difference between men’s and women’s knowledge of modern lifestyle. Gendered deviations between item ratings suggest a link between gender and forms of preferred wealth. Specifically, several items related to relational wealth were rated higher by men than women and in deviation from the consensus answer key. Whether this is a real effect or just an artifact of the exploratory methods used, is an empirical question for future research. That said there is good evidence from other sources that demonstrate similarly gendered differences in determinants of social status, livelihood choices, individual agency and areas of emic knowledge among Tsimane’ men and women.

Finally, the study findings help to fill in gaps in knowledge on Tsimane’ women and social status. The dearth of previous research on women has raised several questions about our general understanding of Tsimane’ social status, central among them being whether previous findings on male social status can be directly interpreted or if they suffer from biases such as the omission of gendered variation in hypothesis testing [99]? As Ioannidis has convincingly argued [100], one of the greatest threats to the validity of any conclusion is the systematic omission of essential variables. For this reason, the findings I describe here should encourage a cautious approach to interpretation of the existing research on Tsimane’ social status. To address concerns about omitted variable bias, future research must investigate the relationship between social determinants and emic lifestyle among a large and gender diverse sample of Tsimane’.
